# Sensitization and Desensitization in Vascularized Composite Allotransplantation

**DOI:** 10.3389/fimmu.2021.682180

**Published:** 2021-08-11

**Authors:** Dimitrios Moris, Linda C. Cendales

**Affiliations:** Department of Surgery, Duke University Medical Center, Durham, NC, United States

**Keywords:** vascular composite allotransplantation, sensitization, desensitization, antibody-mediated rejection, hand transplantation, face transplantation, burns

## Abstract

Vascularized composite allotransplantation (VCA) is a field under research and has emerged as an alternative option for the repair of severe disfiguring defects that result from severe tissue loss in a selected group of patients. Lifelong immunosuppressive therapy, immunosuppression associated complications, and the effects of the host immune response in the graft are major concerns in this type of quality-of-life transplant. The initial management of extensive soft tissue injury can lead to the development of anti-HLA antibodies through injury-related factors, transfusion and cadaveric grafting. The role of antibody-mediated rejection, donor-specific antibody (DSA) formation and graft rejection in the context of VCA still remain poorly understood. The most common antigenic target of preexisting alloantibodies are MHC mismatches, though recognition of ABO incompatible antigens, minor histocompatibility complexes and endothelial cells has also been shown to contribute to rejection. Mechanistically, alloantibody-mediated tissue damage occurs primarily through complement fixation as well as through antibody-dependent cellular toxicity. If DSA exist, activation of complement and coagulation cascades can result in vascular thrombosis and infarction and thus rejection and graft loss. Both preexisting DSA but especially *de-novo* DSA are currently considered as main contributors to late allograft injury and graft failure. Desensitization protocols are currently being developed for VCA, mainly including removal of alloantibodies whereas treatment of established antibody-mediated rejection is achieved through high dose intravenous immunoglobulins. The long-term efficacy of such therapies in sensitized VCA recipients is currently unknown. The current evidence base for sensitizing events and outcomes in reconstructive transplantation is limited. However, current data show that VCA transplantation has been performed in the setting of HLA-sensitization.

## Introduction

Vascular composite allotransplantation is an evolving field in organ transplantation since it has emerged as a viable option to repair tissue defects resulting from traumatic or other injuries in selected patients (1). Vascularized composite allografts (VCAs) consist of anatomically distinct tissues such as skin, muscles, connective tissue, bones and neurovascular elements that are transplanted as a single unit (2–4). So far, VCAs have been used in various settings including transplantation of face, upper or lower extremity, abdominal wall and genitourinary organs (4, 5) (1). As with other solid organ grafts, they are limited by immune mediated rejection and a concomitant requirement for immunosuppression (2–4). Also, candidates for VCA are frequently sensitized, making them susceptible for antibody-mediated rejection (AMR).

Sensitization consists of the ability of the immune system to recognize and react to foreign human leukocyte antigens (HLA) by producing antibodies and developing memory cells, which are common risk factors for acute allograft rejection. In VCA, many possible reasons for sensitization have been described, including blood transfusions, previous pregnancies or transplants as well as cadaveric skin allotransplantation that is commonly used to provide temporary coverage in burn patients. Despite initial reports underestimating the role of antibodies in VCA damage, it is currently established that AMR is also an important process affecting graft viability (5, 6). Thus, many patients are currently precluded for a life-enhancing VCA due to sensitization and lack of well-established desensitization protocols.

We hereby provide an overview on current evidence of sensitization in the field of VCA, followed by posttransplant strategies of desensitization and their potential impact on future management of VCA patients.

## Background of Allo-Sensitization and VCA Specific Considerations

The exposure of the immune system to non-self HLA may result in the generation of HLA antibodies that happens the settings of transfusion, transplantation or pregnancy. The degree of polymorphism in the HLA system results in a large number of non-self stimuli for antibody development (7). In the setting of transplantation, the presence of donor-specific HLA antibodies (DSA) is well-known to be related to hyperacute rejection (8). Kidney transplant literature supports that both pre-existing DSA and DSA produced *de novo*, which appear in the period after 3 months post transplantation (dnDSA) are harmful, although it seems that AMR patients with preexisting DSA had superior graft survival to patients with dnDSA (9). Similarly, DSAs directed against either class of HLA antigen are harmful but is seems that DSAs directed against HLA class II antigens have been more strongly associated with late-onset AMR, *de novo* antibody production, and reduced graft survival (10).

There is significant body of knowledge coming from the burn literature on the mechanisms of sensitization in VCA (**Table 1**). Burn patients experience sensitization (development of anti-HLA antibodies), during resuscitation and wound coverage. Of interest, burn patients are at higher risk for sensitization during resuscitation with blood products and VCA (measured by average panel-reactive antibody; PRA) compared to burn patients undergoing blood transfusion only. Importantly, burn patients can develop higher PRA levels compared to trauma (non-burn) patients (11). In the same setting, a recent study showed that almost all burn patients undergoing resuscitation with blood products and skin allotransplantation developed anti-HLA antibodies, of which about 50% had complement-fixing antibodies. Of interest, the majority of these patients (62%) were considered highly sensitized (PRA≥85%). Cryopreserved, but not glycerol-preserved skin allografts, history of pregnancy, and number of blood units were associated with HLA sensitization (12). Similarly, it was shown that burn patients with skin allografts developed lower PRAs when evaluated during the acute phase of trauma compared to burn skin transplant recipients when tested years after transplant (6 ± 12% *vs* 42 ± 33%, P = 0.08). The latter demonstrates that detection of HLA antibody is lower in the acute burn period than months to years after injury thus increasing sensitization may ultimately limit burn patients’ candidacy for VCA or decrease success of these procedures (13). Some have proposed emergency VCAs in burn patients as potential strategy for early definitive reconstruction avoiding procedures triggering HLA antibody formation (14). The prevalence of sensitization in patients awaiting VCA is unknown relative to other transplants. Trauma patients waiting for hand or face VCA, without extensive transfusion requirement or prior skin transplant, are mostly healthy, young individuals with a low risk of pre-existing sensitization. According to the literature, more than 80% of the patients who have received reconstructive VCAs (hand or face) are male with an average age of about 30 years (15, 16). On the contrary, the average age for kidney transplantation is above 45 years with more than 60% being males (17).

**Table 1:** Sensitizing factors in VCA.

Sensitizing Factors
Burns
Multiple Blood Transfusions
Pregnancy
Previous Transplants
Allogeneic Skin Grafts
Ventricular Assist Devices
Extracorporeal Membrane Oxygenation

## Characteristics of Antibody-Mediated Rejection

Diagnostic criteria for AMR were first described in the setting of kidney and cardiac allografts (5, 18), and subsequently extended for pancreas, liver and lung (19–21). Characteristics of AMR in small intestine and VCA have also been described (22, 23), but consensus criteria for AMR in these organs are lacking. Main universal characteristics of acute AMR include serological evidence of antibodies, histological evidence of endothelial cell injury, complement activation, and infiltration of innate immune cells (24). As far as the allograft endothelium is concerned, it seems that it plays an active role in the pathogenesis of rejection due to its phenotypic changes according to the microenvironment conditions created by post-transplant inflammation, alloreactive lymphocytes, DSA and complement activation, that in turn, might lead to promotion of proinflammatory alloresponses favoring the expression of Th1 T cells, M1 macrophages and NK cells (25). In VCA, if preformed antibodies (DSA) exist, activation of complement and coagulation cascades can result in vascular thrombosis and infarction and thus hyperacute rejection and graft loss. This hypothesis was confirmed again by recent evidence of AMR in highly sensitized face transplant recipients (26, 27). On the contrary, the effects of dnDSA on VCAs are largely unknown. Grafts with potentially high immunogenicity such as VCA may increase the development of dnDSA and the majority of studies have reported that the presence of DSA is associated with rejection and graft impairment (28). Histopathologic assessment of VCAs is critical for the early and accurate diagnosis of rejection and timely institution of effective immunotherapeutic regimens. Currently, AMR is not included in the BANFF classification of VCA rejection (29). Supportive data for AMR have been limited to demonstration of C4d deposition in hand transplant recipients, experience that is currently different to the one from solid-organ transplantation where C4d deposition is commonly associated with DSA (∼40–60%) and is part of the diagnostic criteria for classic AMR. Reports of VCA recipients with C4d deposition had absence of DSA (6, 30, 31). Findings that were partially confirmed by Petruzzo et al. where detected DSA was not related to C4d deposition. However, there has been one confirmed case of AMR in which C4d deposition was specific to the allograft and occurred in the presence of DSA (32).

## Prevention and Management of AMR in VCA

Prevention and management of AMR in VCA is a research area requiring further attention. Currently, prevention and desensitization protocols as well as treatment of AMR in VCA recipients is based on those recommended in solid organ transplantation. In general, despite the understandable advantage of reducing maintenance immunosuppression, with or without cell-based therapy, overly aggressive minimization is potentially linked to a higher incidence of acute rejection episodes, an increased risk of chronic rejection and the development of dnDSA (33). Regarding induction therapy in VCA, antithymocyte globulin (ATG) is currently the most commonly used T-cell depleting induction agent. Other alternative approaches include the use of alemtuzumab and basiliximab (34). For maintenance immunosuppression, protocols commonly used are derived from solid-organ experience and mainly consist of triple therapy with tacrolimus, MMF and steroids (4, 35), There have been reports of management of VCA patients with dual maintenance immunosuppression regimen subsequent to ATG induction (36). Tacrolimus is the most commonly used calcineurin inhibitor with early (initial period of 1–5 months after transplantation) trough levels of 10–15 ng/mL and 5–10 ng/mL thereafter. Tacrolimus trough levels<5ng/mL appeared to be associated with a higher risk for acute rejection (37). Most centers taper steroids rapidly in the early post-transplant period with a subsequent maintenance of 5 to 15 mg/d for 6 to 12 months in most patients (38). Experimental and clinical data also support the preventive role of belatacept as centerpiece of immunosuppressant for VCA by providing sufficient protection against rejection (4, 39–43).

Treatment against AMR usually includes steroids, total plasma exchange, IVIG, plasmapheresis, bortezomib and anti-CD20 mabs (44). Both preexisting DSA but especially *de-novo* DSA are currently discussed as main contributors to late allograft injury and graft failure (7). The Innsbruck group reported the first case of a primarily B-cell-driven rejection episode with the development of dnDSA indicative of AMR in a patient after forearm transplantation at 9 years post-transplant, without recent trigger such as surgery or blood transfusion. The patient did not improve with steroid treatment, but administration of rituximab resulted in complete remission of clinical symptoms (45). A possible explanation for the development of dnDSA in VCA patients might be the association between post T-cell mediated rejection dnDSA development and pretransplant sensitizing events which was not specific to the DSA first detected in the early posttransplant period. It could be possible that most DSA reported as *de novo* are actually secreted by memory B cells undergoing clonal expansion triggered by the proinflammatory microenvironment of T-cell mediated rejection (28, 46). Another major finding in these patients is the evidence of lymphoid neogenesis in the dermis of both grafts reminiscent of tertiary lymphoid organs (47). However, diagnosis of AMR remains incompletely described, as staining for C4d and DSA titers has been shown to be unreliable in VCA (5, 48, 49).

## Desensitization Strategies in VCA

**Figure 1:**
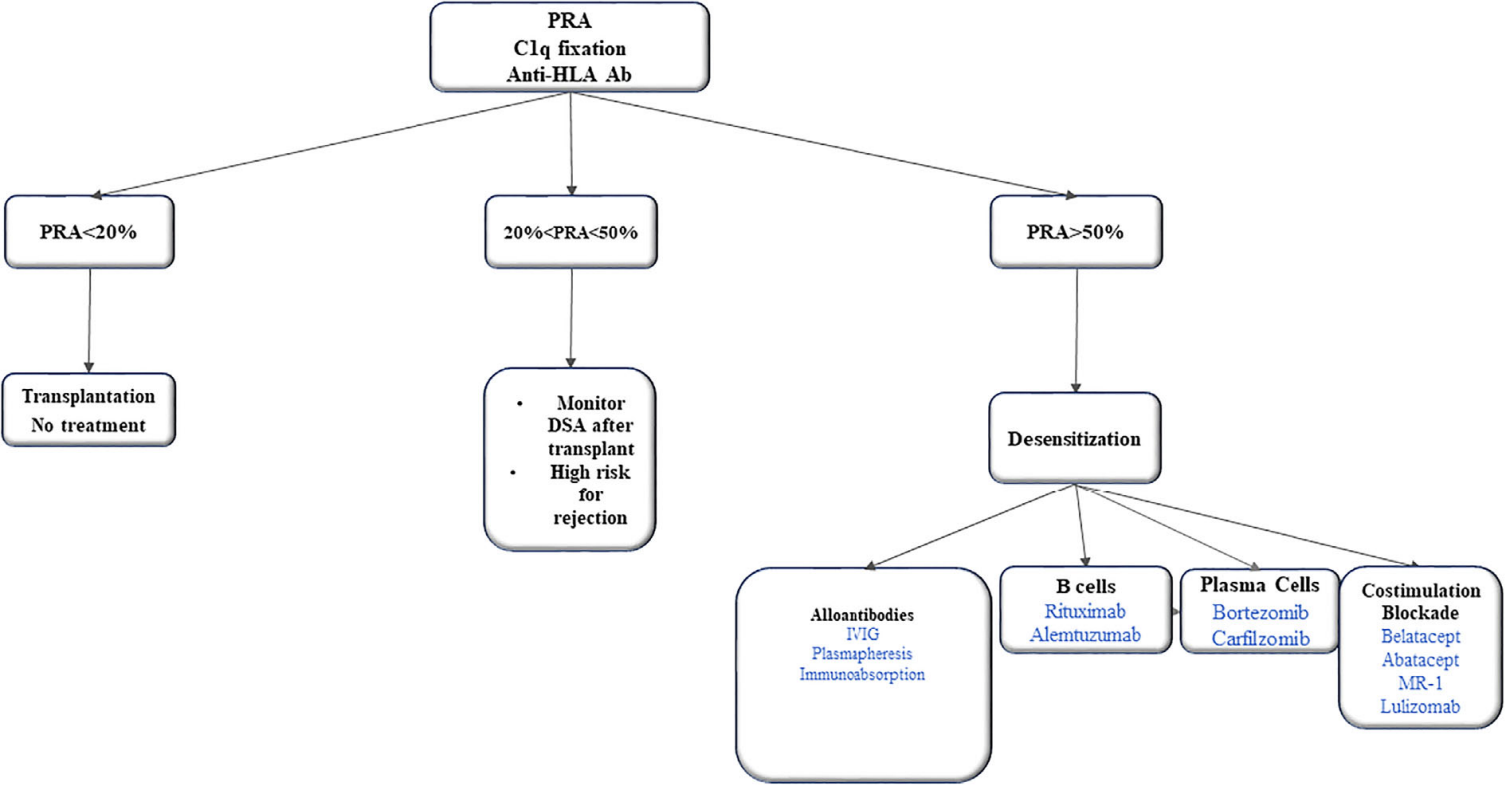
Desensitization strategies in VCA.

There are no well-established desensitization protocols in VCA literature. Recent literature summarized the potential strategies for desensitization in patients with VCA (50, 51). Immunoadsorption and plasma exchange aims to remove, selectively or not, antibodies for antigens A and B. However, antibody titres bounce back a few weeks after treatment if not combined with another treatment. Another treatment is rituximab that deplete B cells. But it does not target plasma cells due to lack of CD20 receptors. The proteasomal inhibitor bortezomib triggers the apoptosis of plasma cells and reduces the alloantibody production *via* this pathway. Intravenous immunoglobulins (IVIG) neutralize anti-idiotypic antibodies, inhibit the complement cascade and reduce antibody formation by down regulating mechanisms or eliciting apoptosis of B cells **(** **Figure 1** **)**.

Many novel data are emerging from experimental animal models and limited human case series. It was recently shown that hindlimb transplant rats with prior skin transplant sensitization showed prolonged graft survival when desensitized with of total body irradiation, fludarabine, and syngeneic hematopoietic stem cell transplantation, that was related to significantly reduced DSA level as well as no evidence of CD4d deposition at the time of rejection (52). In 2014, Chandraker et al. reported the first experience of a full-face allotransplant in a pre-sensitized burn patient with a positive perioperative crossmatch and high levels of circulating anti-HLA class I and class II antibodies with a calculated PRA score of 98. Despite plasmapheresis in addition to induction with ATG, the recipient developed an AMR with rising DSA titers and evidence of C4d positivity in the biopsy, which showed a Banff grade III rejection. The patient received anti-AMR therapy combining plasmapheresis, eculizumab, bortezomib and alemtuzumab. The DSA levels decreased, clinical condition improved and the histological signs of rejection had resolved by 6 months after the transplantation (30). The long-term efficacy of such therapies in sensitized VCA recipients is currently unknown. Whether desensitization strategies will increase the recipient pool of VCA patients remains to be seen. Since the report, the patient experienced irreversible rejection, graft loss and was re-transplanted in July 2020.

Most of the literature on desensitization protocols emerges from kidney transplantation (30, 53). Emerging data from experimental models showed that multiple factors such as proteasome inhibitors, costimulation blockades, BAFF/APRIL blockades and complement inhibitors significantly prolong graft survival by disorganizing germinal center responses and reducing DSA levels (54–56). The idea of using these protocols might be very promising, but there remain pros and cons with these approaches as they have not been totally effective in solid organ transplants and they are accompanied by side-effects such as increasing risk for severe infections, renal failure, thrombotic events and malignancy (28, 57).

## Conclusions

The prevalence of sensitization in patients awaiting VCA is unknown relative to other transplants. Patients qualifying for skin containing VCAs after severe burns who require aggressive resuscitation with multiple blood products and temporary skin coverage are usually at risk of sensitization. The management of potential VCA patients starts at the time of initial injury. The prevention of sensitization and the possible desensitization strategies to extend VCA survival is an area under research. Currently, there is no well-established desensitization protocol for VCA patients. Emerging knowledge from other solid organ transplants might guide management of sensitized VCA patients in the future.

## Author Contributions

All authors listed have made a substantial, direct, and intellectual contribution to the work, and approved it for publication.

## Conflict of Interest

The authors declare that the research was conducted in the absence of any commercial or financial relationships that could be construed as a potential conflict of interest.

## Publisher’s Note

All claims expressed in this article are solely those of the authors and do not necessarily represent those of their affiliated organizations, or those of the publisher, the editors and the reviewers. Any product that may be evaluated in this article, or claim that may be made by its manufacturer, is not guaranteed or endorsed by the publisher.
